# The desert gerbil *Psammomys obesus* as a model for metformin-sensitive nutritional type 2 diabetes to protect hepatocellular metabolic damage: Impact of mitochondrial redox state

**DOI:** 10.1371/journal.pone.0172053

**Published:** 2017-02-21

**Authors:** Inès Gouaref, Dominique Detaille, Nicolas Wiernsperger, Naim Akhtar Khan, Xavier Leverve, Elhadj-Ahmed Koceir

**Affiliations:** 1 Bioenergetics and Intermediary Metabolism team, Laboratory of Biology and Organism Physiology, Biological Sciences Institute, University of Sciences and Technology Houari Boumediene, BP 32, ElAlia, Algiers, Algeria; 2 Université de Bordeaux, Rhythmology and Heart Modeling Institute, Bordeaux, France; 3 CarMeN Laboratory, INSERM U1060, Villeurbanne, France; 4 Physiologie de la Nutrition & Toxicologie, INSERM U1236, Université de Bourgogne Franche-Comté (UBFC), Dijon, France; 5 University Grenoble Alpes, Laboratoire de Bioénergétique Fondamentale et Appliquée (LBFA), INSERM, U1055, Grenoble, France; Medical Clinic, University Hospital Tuebingen, GERMANY

## Abstract

**Introduction:**

While metformin (MET) is the most widely prescribed antidiabetic drug worldwide, its beneficial effects in *Psammomys obesus (P*. *obesus)*, a rodent model that mimics most of the metabolic features of human diabetes, have not been explored thoroughly. Here, we sought to investigate whether MET might improve insulin sensitivity, glucose homeostasis, lipid profile as well as cellular redox and energy balance in *P*. *obesus* maintained on a high energy diet (HED).

**Materials and methods:**

*P*. *obesus* gerbils were randomly assigned to receive either a natural diet (ND) consisting of halophytic plants (control group) or a HED (diabetic group) for a period of 24 weeks. MET (50 mg/kg per os) was administered in both animal groups after 12 weeks of feeding, i.e., the time required for the manifestation of insulin resistance in *P*. *obesus* fed a HED. Parallel *in vitro* experiments were conducted on isolated hepatocytes that were shortly incubated (30 min) with MET and energetic substrates (lactate + pyruvate or alanine, in the presence of octanoate).

**Results:**

*In vivo*, MET lowered glycemia, glycosylated haemoglobin, circulating insulin and fatty acid levels in diabetic *P*. *obesus*. It also largely reversed HED-induced hepatic lipid alterations. *In vitro*, MET increased glycolysis but decreased both gluconeogenesis and ketogenesis in the presence of glucogenic precursors and medium-chain fatty acid. Importantly, these changes were associated with an increase in cytosolic and mitochondrial redox states along with a decline in respiration capacity.

**Conclusions:**

MET prevents the progression of insulin resistance in diabetes-prone *P*. *obesus*, possibly through a tight control of gluconeogenesis and fatty acid β-oxidation depending upon mitochondrial function. While the latter is increasingly becoming a therapeutic issue in diabetes, the gut microbiota is another promising target that would need to be considered as well.

## Introduction

The epidemic nature of type 2 diabetes mellitus (T2DM) has led to its recognition as an urgent priority by the International Diabetes Institute. Indeed, the prevalence of this disease is predicted to reach 5.4% of the worldwide adult population by the year 2025, i.e., 300 million individuals [1]. T2DM is a multifactorial disease. It results from the interaction of environmental factors and genetic predisposition leading to two major abnormalities: insulin resistance and defective pancreatic β-cell function [2]. During the long-lasting phase that precedes the onset of T2DM, hyperinsulinemia compensates for insulin resistance. Hyperglycaemia then develops with a progressive β-cell failure, but the mechanisms involved remain unknown.

Among the oral antidiabetic agents used for the management of T2DM, Metformin (MET) is mostly considered as first-line drug therapy for patients [3]. Besides its action on glucose homeostasis, which is achieved through a potent reduction of hepatic glucose production due to the inhibition of gluconeogenesis, MET also exerts beneficial effects on the blood lipid profile as well as hepatic steatosis in overweight subjects [4] and *ob/ob* mice [5]. It is established that phosphorylation of acetyl-CoA carboxylase by AMP-activated protein kinase (AMPK), a master sensor for the fine tuning of cellular energy requirements, is essential for part of these MET-induced improvements [6]. In addition, the internalization of MET was necessary for its biological action, and its effects were specific on insulin signaling pathway [7]. An important breakthrough for understanding the mode of MET action was evidenced by the suppression of glucose output in hepatocytes *via* AMPK activation [8]. This activation was shown to occur independently of a change in adenylate energy charge [9]. Nevertheless, not all of the MET therapeutic action could be explained by AMPK-dependent mechanisms [10], and there exists now a consensus that MET can activate AMPK due to energetic stress resulting from inhibition of mitochondrial oxidative phosphorylation [11].

Conventional animal models have been employed to elucidate the pathogenesis of T2DM, but they do not recapitulate accurately human disease. In this context, the desert gerbil *Psammomys obesus* (*P*. *obesus*) constitutes a unique model of human android obesity and T2DM [12]. In its native arid environment, the low caloric chenopodiaceae-dominated desert plants serves as its primary diet, but *P*. *obesus* with innate insulin resistance usually remains fatty. This feature is mainly explained by a low hydrolysis of liver glucose-6-phosphate [13], leading to normoglycemia and enhanced lipogenesis. When *P*. *obesus* are transferred to laboratory conditions and fed a HED *ad libitum*, the delicate homeostasis of the gerbil becomes impaired and the combination of (i) hyperinsulinemia together with insulin resistance, (ii) increased lipid storage inducing obesity and non-alcoholic steatohepatitis [14], and (iii) hyperglycemia, is responsible for the detrimental effects on β-cells ultimately leading to ketoacidosis [15]. In diabetic *P*. *obesus*, peripheral insulin resistant state was evidenced by a reduced capacity of insulin to activate hormonal receptor tyrosine kinase [16], and the resulting high rate of hepatic gluconeogenesis was characterized by stimulation of insulino-dependent cellular pathways involving phosphoenolpyruvate carboxykinase, glucose 6-phosphatase and pyruvate dehydrogenase [17]. We recently demonstrated that hepatic deterioration, with high oxidative stress and mitochondrial dysfunction, contributed to deleterious outcomes of insulin resistance in diabetic *P*. *obesus* [18]. Furthermore, food restriction in *P*. *obesus* reversed the symptoms of frank diabetes without reducing insulin secretion [19]. In the same way, the administration of flavonoid silibinin to obese gerbils provided substantial protection against the progression toward metabolic syndrome by blocking the oxidative process and improving liver steatosis [20].

This current study, using both animal and cellular models, was designed to evaluate the impact of MET on plasma and hepatic profiles as well as metabolic abnormalities and energy homeostasis in *P*. *obesus* either developing or not diet-induced experimental diabetes.

## Materials and methods

### Animals, diets, and experimental design

This study involved one hundred adult male gerbils (*Gerbillidae* family, *Psammomys obesus* species) captured in the Algerian Eastern Sahara. Only animals older than 8 months and weighing between 80 and 120 g were included in the experimental protocol. After being examined by a veterinarian (to ensure that the animals were free of diseases), *P*. *obesus* were then transferred to the animal house that was maintained under controlled conditions (22–26°C, 60 ± 10% relative air humidity and 12:12 hours light and dark cycles). They were housed in individual cages with free access to their natural food (ND) that consisted of halophilic plants, primarily belonging to the Chenopodiaceae family (*Traganum nudatum*, *Salsola foetidia*, *Suaedia mollis and Artriplex halimus* species). In this study, we used a *Salsola foetida*-based herbal diet directly harvested from the Algerian North Eastern Sahara, in the area of Biskra (34°25’ North latitude, 5°55’ West longitude). The ND had a very low calorie content of 0.4 kcal/g (80.8% water, 8.4% carbohydrates, 6.9% ash, 3.5% proteins, 0.4% lipids) and was high in dietary fiber (0.18% total sugar, 1.12% lignin, 2.62% hemicellulose, 2.23% cellulose, 2.62% undetermined substances). A complete analysis of the *Salsola* plants showed high levels of various mineral salts (11.8% sodium, 3.8% potassium, 1% calcium, 2.1% magnesium, 3.8% chlorine, 0.2% phosphorus); this composition was standardized by chemical analysis (Caen University, CNRS Research Centre, U405, France) [21]. Following a 2 week-initial accommodation, the gerbils were randomly placed into two experimental groups (n = 30 per batch): the control group consuming their ND and the *P*. *obesus* group eating a HED of 3.25 kcal/g (47% carbohydrates, 25% proteins, 7.5% fat), with high energy carbohydrates (33.5% starch and 13.5% total sugar) and fatty acids (0.81% palmitic acid, 0.05% palmitoleic acid, 0.20% stearic acid, 3.76% oleic acid, 2.40% linoleic acid, 0.28% linolenic acid). Although the latter diet is the standard synthetic food for all laboratory rodents, it is considered to be high caloric (compared to ND) for *P*. *obesus* which subsequently develops diabetes [12]. Food and water consumption were measured daily. Each group was separately subjected to MET treatment that started once HED fed *P*. *obesus* exhibited blood glucose levels > 90 mg/dL (5 mM). *P*. *obesus* were deprived of food 12 h prior to the beginning of the experiment, while they were not deprived of water. Blood samples from the retro-orbital venous plexus were performed weekly in EDTA tubes to determine the fasting plasma parameters. MET treatment commenced on week 12 and continued until the end of the experimental period at week 24. Therefore, the duration of MET treatment in both *P*. *obesus* groups was 12 weeks. Fresh solutions of MET (Merck-Lyon, France) were prepared daily and administrated by gastric intubation at 50 mg/kg of body weight, a quantity within the clinical dose range [22]. Isotonic saline solution (0.9% NaCl) served as the control or placebo treatment. The food intake was evaluated based on the weight of containers at 2, 4, and 6 h after MET administration, and the body weight was determined twice weekly. Body mass index (BMI) of *P*. *obesus* was assessed by dividing the weight (g) by the height (cm) squared. The tail of the animal was not taken into consideration for the BMI calculation.

All the experimental procedures, including *P*. *obesus* capture in the South Eastern Algerian Sahara, were authorized by the Institutional Animal Care Committee of the National Administration of the Algerian Higher Education and Scientific Research (DGRSDT; http://www.dgrsdt.dz). The permits and ethical rules were achieved according to the Executive Decree n°10–90 completing the Executive Decree n°04–82 of the Algerian Government, establishing the terms and approval modalities of animal welfare in animal facilities. The investigation strictly conforms to the *Guide for the Care and Use of Laboratory Animals* [DHHS Publ. No. (NIH) 85–23, revised 1996, Office of Science and Health Reports, Bethesda, MD 20892].

### Blood metabolic parameters analysis and biochemical assays

Plasma glucose levels were measured by the glucose oxidase test combination (Boehringer, Meylan, France). Circulating insulin concentrations were determined by a radioimmunoassay using human insulin as the standard, while ketone bodies, triglycerides and total cholesterol were enzymatically assayed. Plasma lactate concentrations were measured by spectrophotometry and non-esterified fatty acids (NEFA) were determined by microfluorimetry. Glycosylated haemoglobin (HbA1c) was measured by turbidimetry using a Cobas Mira Plus automatic analyser (Roche Diagnostic Systems, Basel, Switzerland). The hepatic content in triglycerides, esterified cholesterol and free cholesterol was carried out by a sequential quantitative method [23]. Glycogen was extracted from tissue samples in KOH at 100°C, assayed after acid hydrolysis according to the method of Chan and Exton [24]. The homeostasis model assessment of insulin resistance (HOMA-IR) was calculated at both baseline and end of the study using the following formula: fasting plasma glucose (mM) x fasting insulin (mU/L) / 22.5 [25]. All the enzyme reagents were procured from Roche (Meylan, France). Lactate, octanoate, pyruvate and alanine were purchased from Janssen Cosmetics (Aachen, Germany). Bovine serum albumin (BSA) was purchased from Sigma chemical Co (St Louis, Missouri, USA).

### Metabolic fluxes, intracellular metabolites and adenine nucleotides analysis

16 h-fasted *P*. *obesus* were anesthetized by intraperitoneal injection of sodium pentobarbital (5 mg/100 g), and hepatocytes were then isolated according to the method of Berry and Friend [26], as modified by Groen [27]. Liver cells were resuspended in Krebs-Ringer bicarbonate buffer (120 mM NaCl, 4.8 mM KCl, 1.2 mM KH_2_PO_4_, 1.2 mM MgSO_4_, 24 mM NaHCO_3_, 2.4 mM CaCl_2_, 2% BSA, pH 7.4), saturated with O_2_/CO_2_ (95:5%). Using a shaking water bath, isolated hepatocytes (10 mg dry cells per mL) were incubated for 30 min at 37°C in closed vials containing 2.5 mL of oxygenated buffer with alanine (20 mM) or lactate + pyruvate (10:1 mM) in the presence of medium-chain fatty acid octanoate (2 mM), with or without MET (1 mM and 10 mM). After 30 min, 350 μL of the cell suspension were deproteinized with HClO_4_ (5% final), then centrifuged at 13,500x*g* for 5 min. The supernatants were neutralized with KOMO buffer (2 M KOH, 0.3 M MOPS) for subsequent assays of glucose, lactate, pyruvate and ketone bodies (3-hydroxybutyrate, acetoacetate) by spectrophotometry [28]. A second sample from the vial was used for the determination of nucleotide content in mitochondrial and cytosolic fractions after separation of cells by a quick spin across a silicone oil layer. ATP and ADP were measured by high performance liquid chromatography as described earlier [29].

### Determination of oxygen consumption rates

After incubation of hepatocytes with substrates, in the presence or absence of MET as described above, the cell suspension was quickly saturated with O_2_/CO_2_ and immediately transferred into a stirred vessel equipped with a Clark oxygen electrode. The oxygen consumption rate was measured at 37°C and expressed as μmol O_2_/min/g dry cell.

### Data analysis

Statistical analysis was performed using SPSS version 12 (SPSS UK, Surrey UK). Data were expressed as the mean ± SEM, and comparisons between groups were made using student's *t* test or one-way analysis of variance (ANOVA) as appropriate. The differences were considered statistically significant at p < 0.05.

## Results

### Effects of MET on BMI, caloric intake and plasma metabolic profile

Adult *P*. *obesus* ate over 30.4 ± 2.8 kcal/day (estimated to be around 76 g of food daily or 80% of their body weight) when maintained on an exclusive ND. In such conditions, these rodents do not exhibit hyperglycemia, hyperinsulinemia or plasma lipid impairments. In contrast, the caloric intake was markedly increased in HED fed *P*. *obesus* (312 ± 8 kcal/day), and their body weights almost doubled when compared to the control group (Table 1).

**Table 1:** Caloric intake, body weight and plasma metabolic profile in control (ND) and diabetic (HED) *P*. *obesus* exposed or not exposed to metformin.

Parameters/Groups	Control (ND)	Placebo (ND+MET)	Diabetic (HED)	Diabetic (HED+MET)
Caloric intake (cal/100 g wet weight)	30.4±2.8	25.9±1.7	312±8	124±3***
Body weight (g)	76±5	73±1	145±3	109±5**
BMI (g/cm2)	0.35±0.01	0.28±0.01	0.67±0.03	0.42±0.02*
HbA1c (mmol/mol)	18.8±0.75	11.9±0.65*	67.5±1.05	21.5±0.85***
Glucose (mM)	3.22±0.41	3.11±0.18	15.2±1.4	3.88±0.17***
Insulin (pM)	130±21	121±17	580±47	110±14***
HOMA-IR	2.57±0.14	2.21±0.09	5.33±0.23	2.68±0.11**
Triglycerides (mM)	0.80±0.34	0.74±0.01	1.60±0.61	0.83±0.5**
Total cholesterol (mM)	1.50±0.30	1.38±0.32	2.80±0.5	1.41±0.2*
HDL-cholesterol (mM)	0.65±0.30	0.59±0.21	1.19±0.04	0.95±0.06
LDL-cholesterol (mM)	0.50±0.40	0.44±0.13	0.97±0.30	0.29±0.2**
NEFA (μM)	639±65	613±15	894±132	627±89**
Lactate (mM)	0.65±0.09	1.05±0.03**	0.93±0.14	1.13±0.25
Ketone bodies (mM)	0.57±0.06	0.48±0.04	1.33±0.20	0.80±0.05**

Metformin (MET) was orally administered for 12 weeks at 50 mg/kg/day. Circulating parameters were assayed in 16 h-fasted *P*. *obesus*, and data are expressed as means ± SEM (n = 30/group).

*p < 0.05

**p < 0.01

***p < 0.001 compared with the corresponding dietary condition without metformin treatment. ND: Natural Diet, HED: High Energy Diet

Interestingly, both parameters were greatly reduced in diabetes-prone *P*. *obesus* after chronic administration of 50 mg/kg of MET. Nonetheless, the weight gain decreased more mildly than the caloric intake, meaning *P*. *obesus* remain obese despite MET treatment. BMI was reduced by 37% (p < 0.05) in MET-treated diabetic *P*. *obesus* compared to untreated diabetic gerbils (Table 1). Moreover, MET did not significantly alter neither the caloric intake nor the body weight of the control animals. As expected, the diabetic *P*. *obesus* developed metabolic syndrome with multiple anomalies. Both fasting plasma glucose and insulinemia were particularly increased. MET administration mainly lowered plasma glucose levels (-74%, p < 0.001) in this group of animals. The decline was progressive throughout the course of the treatment until reaching a stable end-value (3.88 ± 0.17 mM) near the baseline. Concomitantly, MET reduced the amount of HbA1c. The values in plasma glucose and HbA1c were not BMI-dependent (0.50 < or > 0.60 g/cm^2^), since they correlated strongly with HOMA-IR and insulinemia in HED fed *P*. *obesus* (r = 0.951 and 0.806, respectively). This lost of sensitivity to insulin in obese animals, as demonstrated by the higher value of HOMA-IR and subsequent hyperinsulinemia, was fully reversed by MET (Table 1). All of this confirmed the long-recognized efficacy of MET upon glucose homeostasis in such a diabetogenic setting. We suggest that its glucose-lowering effect was due to an inhibition of hepatic gluconeogenesis (see data below on isolated hepatocytes). It is noteworthy that MET increased plasma lactate in control *P*. *obesus* to a level similar to that measured in diabetic *P*. *obesus*, but it did not further aggravate lactate accumulation caused by the HED. Otherwise, MET-treated control animals had a lowered HbA1c compared to baseline (Table 1).

### Effects of MET on lipidemia, ketogenesis and liver metabolic parameters

While no significant impact of MET was found in control *P*. *obesus*, its benefits on lipid profile and ketone bodies production in *P*. *obesus* expressing the pathological phenotype represents another key finding in this work. MET treatment abolished net increases of triglycerides, total cholesterol, LDL-cholesterol and NEFA, without changing HDL-cholesterol levels (Table 1). This potent hypolipidemic effect of MET positively correlated with reduced HbA1c (r = 0.70, p = 0.03) and fasting glucose (r = 0.74, p = 0.01). In addition, MET treatment markedly decreased plasma ketone bodies (-40%, p < 0.01) in diabetic *P*. *obesus*.

Furthermore, a significant twofold increase of hepatic triglycerides along with liver mass increase indicated a severe tissue dysregulation in diabetic *P*. *obesus* (Table 2). This harmful fat accumulation was mitigated under MET treatment, which normalized the liver mass/body weight ratio. MET did not alter the hepatic lipid status when given orally to control *P*. *obesus*, but it increased hepatic glycogen content in the diabetic state *vs*. control group (Table 2). This finding possibly reveals that MET would be able to stimulate the utilization of glucose as glycogen stores in the liver of injured *P*. *obesus*.

**Table 2:** Comparison of hepatic biochemical parameters between ND-fed and HED-fed *P*. *obesus* in the absence or presence of metformin.

Parameters/Groups	Control (ND)	Placebo (ND+MET)	Diabetic (HED)	Diabetic (HED+MET)
Hepatic glycogen (g/100 g wet wt)	2.55±1.0	2.75±1.7	2.68±0.33	3.73±1.72*
Total hepatic lipids (g/100 g wet wt)	3.19±0.10	2.97±0.31	4.03±0.16	3.01±0.66*
Hepatic glycerides (g/100 g wet wt)	0.191±0.05	0.179±0.01	0.653±0.03	0.283±0.03**
Liver mass (% body wt)	3.51±0.31	2.99±0.15	4.03±0.80	2.89±0.20**

*p < 0.05

**p < 0.01 compared with the corresponding dietary condition without metformin treatment.

### Effects of MET on cell energy metabolism and cellular redox states

It is important to recall that diabetic patients on MET treatment display plasma drug concentrations ranging from 10 μM to 40 μM. However, it should also be noted that the liver receives the majority of its blood via the portal vein, which may contain substantially higher amounts of MET [22]. Because of hepatic accumulation, MET concentrations more than 250 μM in the liver of diabetic rodents can be reached after a single dose of 50 mg/kg [30]. To study the impact of MET on metabolic pathways in hepatocytes isolated from *P*. *obesus*, we chose a MET dosing which was more related to the range (upper limit) of *in vivo* tissue concentrations than the observed blood levels in clinical use. MET used at 1 mM or 10 mM does decrease hepatic glucose ouput in diabetic *P*. *obesus*. This dose-dependent effect was observed with different gluconeogenic substrates such as lactate + pyruvate or alanine in the presence of octanoate (Table 3). The basal rate of gluconeogenesis from lactate + pyruvate was higher than that from alanine in HED fed *P*. *obesus*, though MET exerted its inhibitory effect in both substrate conditions (Table 3).

**Table 3:** Effects of metformin on gluconeogenesis, glycolysis and ketogenesis in isolated hepatocytes from all *P*. *obesus* groups.

Metabolic fluxes (μmol/min/g dry cells)	Energy Substrates	Control (ND)	Placebo (ND)		Diabetic (HED)	Diabetic (HED)	
			1mM MET	10mM MET		1mM MET	10mM MET
Glucose production	Ala+Oct	4.53±0.57	4.11±0.71	3.99±0.44	8.27±0.35	2.67±0.11***	1.23±0.16***
	(L+P)+Oct	5.57±0.41	5.23±0.15	5.01±0.66	11.03±0.92	4.93±0.26***	2.76±0.11***
Glycolysis	Ala+Oct	1.85±0.15	2.55±0.09*	3.61±0.05**	3.19±0.25	4.62±0.21**	6.97±0.66***
Ketogenesis	Ala+Oct	4.66±0.13	4.09±0.31	3.97±0.64	6.75±0.71	3.23±0.44***	1.32±0.10***
	(L+P)+Oct	3.78±0.41	3.03±0.10	3.01±0.55	5.48±0.16	2.80±0.12***	2.55±0.17***

Primary hepatocytes from 16 h-fasted *P*. *obesus* were incubated in Krebs/bicarbonate buffer containing alanine (Ala) or lactate + pyruvate (L+P) together with octanoate (Oct), in the absence or presence of metformin (1 or 10 mM). Data are means *±* SEM (n = 15).

*p < 0.05

**p < 0.01

***p < 0.001 compared with the corresponding dietary condition without metformin treatment.

**Fig 1:**
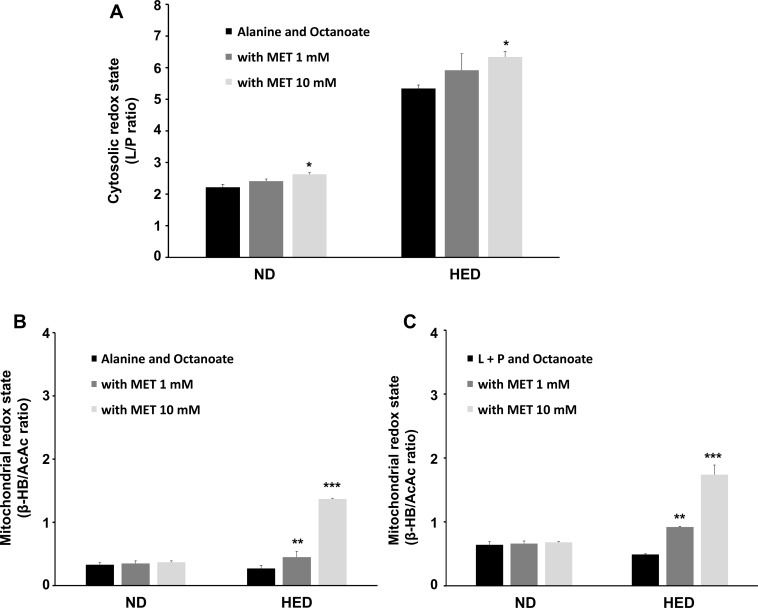
Effect of metformin on both cytosolic and mitochondrial redox states (NADH/NAD^+^) in *P*. *obesus* fed ND or HED. Hepatocytes were incubated for 30 min with energy substrates in the absence or presence of metformin at the indicated concentrations before calculating the lactate/pyruvate ratio (**A**) and β-hydroxybutyrate/acetoacetate (β-HB/AcAc) ratio (**B and C**). Data are means ± SEM of 15 separate experiments. *p < 0.05; **p < 0.01 compared with the group without metformin for each corresponding dietary condition.

**Fig 2:**
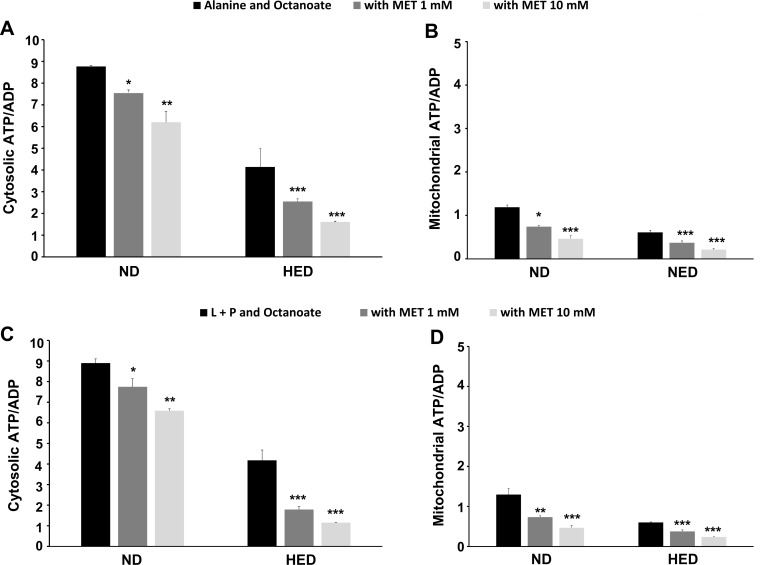
Effect of metformin on both cytosolic and mitochondrial phosphate potential (ATP/ADP) in *P*. *obesus* fed ND or HED. Hepatocytes were incubated for 30 min with alanine + octanoate (**A and B**) or lactate + pyruvate + octanoate (**C and D**) in the absence or presence of metformin at the indicated concentrations, before assaying the content in adenine nucleotides within each compartment. Data are means ± SEM of 15 separate experiments. *p < 0.05; **p < 0.01; ***p < 0.001 compared with the group without metformin for each corresponding dietary condition.

In addition, MET substantially increased the glycolytic flux from alanine as evidenced by the respective 45% and 110% increases with 1 mM or 10 mM MET. As a result, the L/P ratio, which is in thermodynamic equilibrium with cytosolic NADH/NAD^+^, was augmented by 20% in the presence of 10 mM MET (Fig 1A). Of note, MET failed to significantly inhibit gluconeogenesis in control *P*. *obesus*, however the glycolytic pathway was activated but to a lesser extent than in diabetic *P*. *obesus*. After looking at ketogenesis, assessed by the initial oxidation rate of medium-chain octanoate fatty acid, MET did not inhibit the formation of ketone bodies in control animals but fully suppressed their higher production in the diabetic group, regardless of the applied drug concentration (Table 3). The sizeable rise in the β-hydroxybutyrate/acetoacetate ratio, used as a surrogate of the mitochondrial redox potential (Fig 1B and 1C), was consistent with the reductions in either oxygen consumption (Table 4) or cytosolic and mitochondrial ATP/ADP levels in HED fed *P*. *obesus* (Fig 2).

**Table 4:** Effect of metformin on respiratory capacity of isolated hepatocytes from all *P*. *obesus* groups.

Oxygen consumption rate or JO2 (μmol/min/g dry cell)						
Energy Substrates	Control (ND)	Placebo (ND)		Diabetic (HED)	Diabetic (HED)	
		1 mM MET	10 mM MET		1 mM MET	10 mM MET
Ala+Oct	19.60 ± 0.64	14.90 ± 0.15**	11.33 ± 0.51***	10.23 ± 0.85	8.09 ± 0.13**	5.43 ± 0.32***
(L+P)+Oct	23.12 ± 0.97	17.58 ± 0.24**	13.36 ± 0.79***	11.85 ± 0.43	9.37 ± 0.21**	6.29 ± 0.66***

Hepatocytes were processed as in Table 3 before measuring oxygen consumption rates. Data are means *±* SEM (n = 15).

**p < 0.01

***p < 0.001 compared with the corresponding dietary condition without metformin treatment.

These responses were in relation to the aforementioned modifications in glucose metabolism, arguing for the well-known action of MET at these high concentrations on the respiratory chain activity. However, if most of these *in vitro* observations corresponded to *in vivo* data, we cannot omit the emerging role of intestinal microbiota in this pathophysiological context as discussed below.

## Discussion

The results presented in this study show that MET prevented the progression of nutritional diabetes in *P*. *obesus*, a particular strain of herbivorous rodents with a genetic predisposition to develop a metabolic syndrome, and this event was associated with alterations of glycolysis, gluconeogenesis and ketogenesis *in vitro*. The MET-induced changes in liver metabolic fluxes were combined with an inhibition of oxygen consumption, leading to an increased intracellular redox potential and a lower energy charge.

Previous reports, including ours, have shed light on pronounced physiological disorders in *P*. *obesus* after hypercaloric diet feeding [31, 32, 18]. We further extend these observations herein, showing that 24-week HED fed *P*. *obesus* exhibited hyperglycemia, hyperinsulinemia and altered hormonal response as evidenced by a higher index of insulin resistance. A severe dysregulation in blood lipid profile and hepatic fat content characterized more particularly this diabetes-prone *Psammomys* species. Hence, its metabolic-endocrine system was impaired under restrictive laboratory conditions, emphasizing the contribution of lipotoxicity in such adverse environment [33, 34]. *In vivo* MET treatment (from week 12 to week 24) prevented the progression of systemic metabolism disturbances and restored insulin sensitivity in diabetic *P*. *obesus*, as evidenced by a controlled glycemia and a lowered HOMA-IR. In T2DM and/or obese patients, the primary mechanism for MET action consists of selective inhibition of whole body free fatty acid oxidation while leaving the mitochondrial β-oxidation capability unaffected [35]. It was also reported that MET lowered circulating lipids levels by promoting the clearance of VLDL-triglyceride from brown adipose tissue [36]. In MET-treated diabetic *P*. *obesus*, a remarkable improvement of hepatic triglyceridemia and cholesterolemia, in association with a decline in plasma NEFA, was observed. The reduced availability of plasma fatty acids together with other coordinated effects on the liver, possibly on the adipose tissue, and potential other effects were tightly related to a MET-driven enhancement of fat metabolism in these diabetic *P*. *obesus*, thereby alleviating their advanced complications. Indeed, the inhibition of lipids storage is essential to restore both insulin sensitivity and glycemia in *P*. *obesus* fed a regular diet. Importantly, these results were obtained at a dose of MET (50 mg/kg) that was clinically shown to decrease plasma glucose to optimal levels [37].

Using this unique *Psammomys* model, Harmel et al. documented the positive effects of MET on hyperlipidemia associated with insulin resistance and T2DM; these changes began in the small intestine and were dependent on AMPK activity [38]. Similarly, MET activated a duodenal AMPK-dependent pathway that lowered hepatic glucose production in rat models of diabetes [39]. Such observations are parallel to the recent recognition of increased intestinal permeability as a consequence of high fat intake or bacterial modifications in gut microbiota [40–42]. Limited findings have described different microbial communities colonizing the digestive tract of *P*. *obesus* [43, 44]. This intestinal microflora is closely related to the sandy soil where halophyte plants (the dominant food of desert gerbils) grow. The high content in micronutrients and cellulose fibres of this herbal diet can therefore interact with the bacteria in order to modulate the host energy metabolism and physiology. As some bacterial strains possess oxidative capacities [45], we suggest that fermentation of dietary fibres by the gut microbiota of *P*. *obesus* under ND conditions generates short-chain fatty acids (i.e., propionate and butyrate) which should in turn activate intestinal gluconeogenesis, providing a source of glucose during starvation or inducing *de novo* hepatic lipogenesis. Because it was recently established that the bodies of diabetic people have a lower proportion of butyrate-producing bacteria [46], it is likely that both the composition and metabolic functions of the gut microbiota were similarly changed in *P*. *obesus* ingesting a HED. Assuming that a modulation of the gut microbiota by MET constitutes a novel component of its antidiabetic action [47, 48], one can hypothesize, in the light of our overall findings (e.g., key role of fatty acid metabolism in MET-induced improvement of diabetic phenotypes in *P*. *obesus*), that MET may alter the gut microbiota diversity of *P*. *obesus* in parallel with its effects on host (patho) physiology and that the magnitude of this modulatory effect is probably diet-dependent. This hypothesis is highly interesting and asks for further research.

Regarding other issues for MET action, we were struck by the consistent observation of increased plasma lactate amounts in drug-treated animals and patients [49, 50]. It has also been shown that the human intestinal mucosa appears to be an important source of MET-induced lactate production [51], even though delayed glucose absorption still took place more distally along the tract. In our experimental conditions, plasma lactate increased in healthy control *P*. *obesus* after MET exposure but lactatemia did not further worsen in diabetic *P*. *obesus* given MET. The plasma level of ketone bodies however remained rather high, presumably due to an accumulation of glycogen in the damaged liver of *P*. *obesus*. Collectively, our *in vivo* findings suggest the relevance of MET action on the crossroads between lipid and glucose metabolism. This could be partly mediated by intestinal and liver mechanisms without ruling out the implication of circulating redox state since it was found that altered plasma lactate/pyruvate ratios can regulate liver metabolism in mice [52].

Based on *in vitro* results, achieved by contrast with MET concentrations (millimolar) higher than those applied in human therapy, which excluded any role of gut microbiota at the cellular level, we assured that MET ameliorated hyperglycemia in *P*. *obesus* through inhibition of gluconeogenesis from various energizing substrates. Lactate + pyruvate supplied reducing equivalents (NADH_2_, FADH_2_) from glycolysis to the mitochondrial respiratory chain and, without modifying regulation of the metabolic routes; the cytosolic redox potential was imposed using a concentration ratio for these substrates of 10:1 [53]. Alanine is the principal amino acid taken up by the liver during ingestion of a low protein diet or starvation and, as such, is a key precursor for gluconeogenesis [54]. Octanoate, a medium chain fatty acid, provided reducing equivalents to the mitochondria through ß-oxidation, also altering gluconeogenesis in perfused livers [55]. In our experimental conditions, MET inhibited glucose production in a dose-dependent manner, regardless of the glucogenic substrates used (Table 3). Although we did not determine the activity of some key enzymes here, the underlying mechanism could be a lowering in the uptake of substrates by liver cells [56] or an enhanced gluconeogenic flux through pyruvate kinase [57]. Another salient point of this *in vitro* study was the link between ketone body production and fatty acid ß-oxidation in MET-treated diabetic *P*. *obesus*. If the decrease in ketone bodies could be accounted for by a reduction in octanoate oxidation, at least partly (ketogenesis reflecting in fact the flux through fatty acid ß-oxidation), the results were rather in favour of an enhanced ß-oxidation. Owing to the inhibition of gluconeogenesis by MET, the amount of oxaloacetate available for the reaction with acetyl-CoA increases, and acetyl-CoA is then preferentially channelled from entering the Krebs cycle at the expense of the pathway producing acetoacetate and β-hydroxybutyrate. This kind of process can operate because MET acutely inhibited oxygen consumption rates in hepatocytes from diabetic *P*. *obesus*, thereby decreasing ATP content and increasing redox potential. To re-oxide the reducing equivalents in the case of diminished glucose synthesis, MET not only stimulated glycolysis (by increasing lactate) but also enhanced fatty acid β-oxidation concomitantly with an inhibition of ketogenesis. All these statements were consistent with the fact that MET facilitated the removal of plasma NEFA and improved hepatic lipid metabolism in diabetic *P*. *obesus*.

MET was revealed to inhibit mitochondrial glycerol-3-phosphate dehydrogenase, raising the cytosolic NADH/NAD+ ratio and impairing utilization of redox-dependent substrates for gluconeogenesis, but surprisingly the mitochondrial NADH pool decreased [58]. Here as in other reports [59], the result was quite the opposite. MET reduced this intracellular compartment much more as evidenced by the rise in β-hydroxybutyrate/acetoacetate ratio, a common index of mitochondrial redox state. Despite these discrepancies, possibly reflecting the differences in protocol used for drug administration and/or treatment duration, one may stress that the major target of MET was mitochondria since it selectively inhibited respiratory chain complex I [60, 61]. Taking into account that we highlighted the occurrence of increased oxidative stress along with defective oxidative phosphorylation in diabetic *P*. *obesus* [18], the current results warrant further investigation for better deciphering the exact mitochondrial role of MET in this rodent strain. When comparing the metabolic action of MET on hepatocytes from normal Wistar rats with that from control lean *P*. *obesus*, this drug induced mixed effects in the latter. Indeed, gluconeogenesis was barely affected and glycolysis was increased but both pathways were significantly altered in Wistar rats [13]. This may correspond to distinct modes of behaviour, keeping in mind that insulin was relatively high in spite of a very low basal metabolic rate associated with a slow digestion of plants (ND) in the cecum of *P*. *obesus* [62], thus permitting them to stimulate lipogenesis, when the capacity for glucose oxidation was smaller than in Wistar rats [13, 63].

Although some studies found that MET reduced body weight gain in patients suffering from diabetes [64], we did not observe any effect on fat deposition in diabetic *P*. *obesus*. Meanwhile, the caloric intake was substantially lessened by MET. This drug could thus indirectly influence the central nervous system through a modulation of hypothalamic feeding circuits [65]. Indeed, by accumulating into the gut, MET increased incretins level, including glucagon-like peptide-1 (GLP-1), in obese patients [66] and T2DM animal models [67]. In that respect, authors disclosed an upregulation of GLP-1 release in metabolically challenged *P*. *obesus* rats [68]. These last observations reinforce the idea that MET might exert its glucose-lowering action *via* the gut as newly proposed in human beings, at least when administrated orally [69]. An explanation for the reduced food intake seen in diabetic *P*. *obesus* under MET treatment could be linked to this phenomenon but direct evidence for this was not available.

## Conclusions

**Fig 3:**
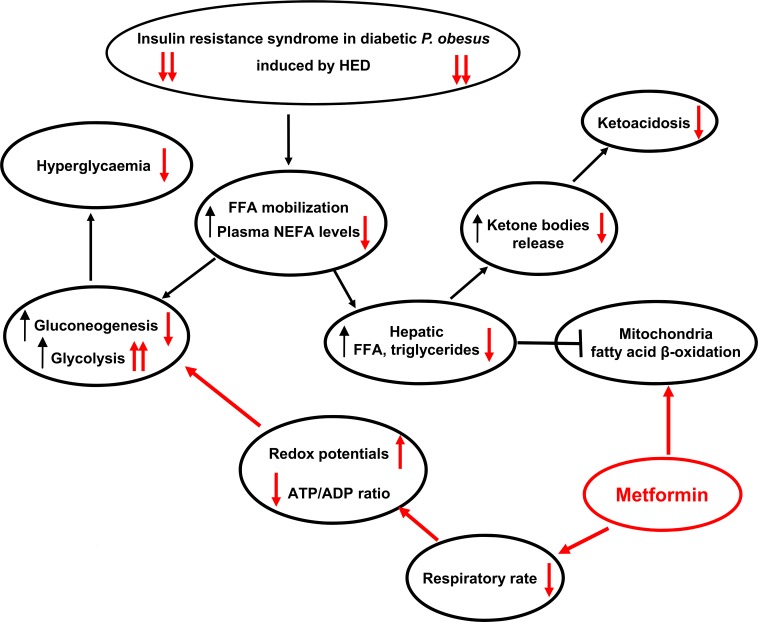
Summary of the metformin effects on diabetic syndrome evolution in *P*. *obesus* fed a HED. *P*. *obesus* is a unique gerbil species predisposed to develop morbid obesity, ingestion of HED induces a high flux of free fatty acids which first activate gluconeogenesis (causing glucose intolerance followed by hyperglycemia), and later become toxic for the animals (leading to dyslipidemia, ketoacidosis and energy deficit). Metformin is then able to hamper this cascade of detrimental events or processes by mainly acting at the intracellular level (*i*.*e*., mitochondria), with positive influence on systemic metabolism homeostasis through improvement of insulin resistance. See the text for details.

If healthy *P*. *obesus* did not fulfill the criteria for metabolic syndrome, by means of adaptation to a hypocaloric food (ND), transferring them to a HED for 24 weeks led to profound metabolic disorders which were acutely corrected by MET. Our *in vivo* and *in vitro* findings confirmed its effectiveness to ameliorate ketoacidosis and lipid metabolism in diabetic *P*. *obesus*; these events likely relied on hepatocellular redox status and mitochondrial activity (see Fig 3 for summary).

This study likewise agreed with the successful use of MET for patients intolerant to glucose or with moderate hyperglycemia. Such data are of utmost importance for establishing innovative therapeutic strategies, and any comparison between the natural history of diabetes in *P*. *obesus* and human pathology should help explain, at least partially, the higher risk of developing morbid obesity for people with unhealthy dietary habits. In addition, part of the MET taken orally by *P*. *obesus* might directly interact with the microbiota of the gastrointestinal tract before entering the circulation and peripheral tissues to protect host cells against severe damages. This open question linked to the dependency and interaction of MET effects with microbiome in metabolic diseases needs to be further elucidated.

## Abbreviations

T2DM: type 2 diabetes mellitus
MET: metformin
AMPK: AMP-activated protein kinase
BMI: body mass index
HbA1c: glycosylated haemoglobin
NEFA: non-esterified fatty acids
HOMA-IR: homeostasis model assessment for insulin resistance
EAK: Elhadj-Ahmed KOCEIR
DD: Dominique DETAILLE
IG: Ines GOUAREF
NW: Nicolas WIERNSPERGER
NAK: Naim Akhtar KHAN
XL: Xavier Leverve
